# Genomic object detection: An improved approach for transposable elements detection and classification using convolutional neural networks

**DOI:** 10.1371/journal.pone.0291925

**Published:** 2023-09-21

**Authors:** Simon Orozco-Arias, Luis Humberto Lopez-Murillo, Johan S. Piña, Estiven Valencia-Castrillon, Reinel Tabares-Soto, Luis Castillo-Ossa, Gustavo Isaza, Romain Guyot

**Affiliations:** 1 Department of Computer Science, Universidad Autónoma de Manizales, Manizales, Colombia; 2 Center for Technology Development Bioprocess and Agroindustry Plant, Department of Systems and Informatics, Universidad de Caldas, Manizales, Colombia; 3 Department of Electronics and Automation, Universidad Autónoma de Manizales, Manizales, Colombia; 4 Institut de Recherche pour le Développement, CIRAD, Univ. Montpellier, Montpellier, France; Ben-Gurion University, ISRAEL

## Abstract

Analysis of eukaryotic genomes requires the detection and classification of transposable elements (TEs), a crucial but complex and time-consuming task. To improve the performance of tools that accomplish these tasks, Machine Learning approaches (ML) that leverage computer resources, such as GPUs (Graphical Processing Unit) and multiple CPU (Central Processing Unit) cores, have been adopted. However, until now, the use of ML techniques has mostly been limited to classification of TEs. Herein, a detection-classification strategy (named YORO) based on convolutional neural networks is adapted from computer vision (YOLO) to genomics. This approach enables the detection of genomic objects through the prediction of the position, length, and classification in large DNA sequences such as fully sequenced genomes. As a proof of concept, the internal protein-coding domains of LTR-retrotransposons are used to train the proposed neural network. Precision, recall, accuracy, F1-score, execution times and time ratios, as well as several graphical representations were used as metrics to measure performance. These promising results open the door for a new generation of Deep Learning tools for genomics. YORO architecture is available at https://github.com/simonorozcoarias/YORO.

## Introduction

Transposable elements (TEs) are DNA sequences that have the ability to move to different locations within the genome and sometimes to multiply their copy number [1]. They have been found in virtually all eukaryotic genomes, comprising up to 80% of genetic material in plants [2, 3]. As a result, they are associated with functional sequence alterations [4], chromosomal rearrangement events, genetic variability, species mutation [5], genome size variation and adaptation to the environment [6, 7].

According to the transposition mechanisms, TEs can be classified into two classes: Class I, also known as retrotransposons, uses a “copy-and-paste” mechanism by utilizing an RNA molecule. Class II or transposons, on the other hand, employs a “cut and paste” mechanism using a DNA molecule as an intermediary [1] and in the specific case of helitrons a “peel and paste” mechanism. These classes can be further divided into subclasses, orders, superfamilies, lineages/families, and subfamilies [8, 9]. Long terminal repeat retrotransposons (LTR-RT) are the predominant repetitive sequences in plant genomes, accounting for up to 75% of nuclear DNA, as in the case of some cereals (wheat, barley, and corn) [10]. Various studies suggest that these elements are closely related to genome size evolution, driving rapid genomic changes and being responsible for the extensive variety of flowering plants [11, 12].

**Fig 1:**
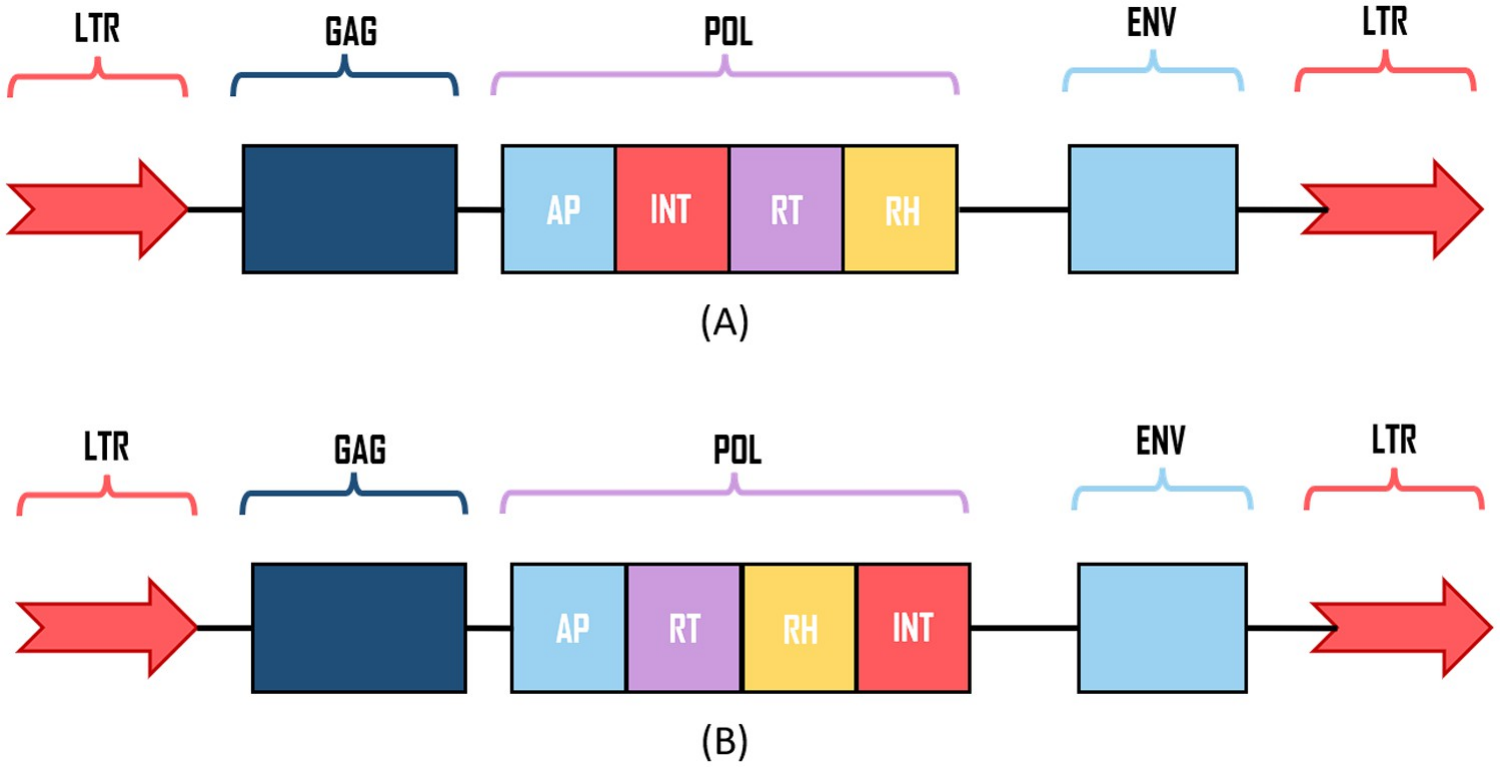
Internal structure and organization of LTR-retrotransposons in plants for: (A) Ty1/copia superfamily and (B) Ty3/gypsy superfamily. Depending on the position of the integrase (INT) domain, the element can be classify to Ty1/copia or Ty3/gypsy superfamily.

Despite the great diversity of LTR-RT that can be observed in nucleotide sequences, they possess a conserved structure that include two long terminal repeats (LTRs) flanking the internal part containing the *gag* and *pol* genes required for the mobility process (Fig 1). The *gag* gene encodes the structural proteins, such as the capsid (CA) and nucleocapsid (NC), while the *pol* gene encodes the proteins comprising the enzymatic group responsible for the transcription process and integration into the new genome location: aspartic proteinase (AP), reverse transcriptase (RT), RNase H (RH), and integrase (INT) [13]. Some elements also present an incomplete/non-functional *env* gene, which is thought to be a reminder of ancient viruses.

Based on the arrangement of the RT and INT domains in the pol gene, LTR-retrotransposons can be classified into the *Ty1/copia*, *Ty3/gypsy*, *Bel-Pao*, retroviruses, and endogenous retroviruses superfamilies. Only the *Ty1/copia* and *Ty3/gypsy* superfamilies are found in plants [8]. These are further classified into lineages/families. Lineages for *Ty1/copia* include *Ale, Alesia, Angela, Bianca, Bryco, Lyco, Gymco-I, II, III, IV, Ikeros, Ivana, Osser, SIRE, TAR* and *Tork*. And the lineages for *Ty3/gypsy* include *CRM, Chlamyvir, Galadriel, Tcn1, Reina, Tekay, Athila, Tat-I,II,III, Ogre, Retand, Phygy* and *Selgy* [11, 12, 14, 15].

The annotation of TEs can be performed by different software, however their execution times are usually a major bottleneck in genomics studies, specially for large genomes. The increasing amount of data required for analysis turns bioinformatics tasks into “Big Data” tasks [16, 17]. Most commonly used tools do not employ massive data processing strategies, and even some processes such as curation are still performed manually by experts in the field [18]. Tools that apply parallelization strategies are not fully scalable (i. e. they do not use CPU+GPU when available, and cannot be executed over distributed memory architectures), making it difficult to analyze large genomes. Also, those tools have a high false positive rate, create redundant and fragmented libraries, which limits confidence in TE studies [19–21].

Deep learning (DL) strategies have been shown to be effective for LTR-RT annotation [22, 23], improving execution time, without affecting their efficiency and sensitivity. The use of deep learning-based strategies allows exploiting of the potential of parallel architectures and GPUs (Graphical Processing Unit), solving the bottlenecks generated by sequential programs or inefficient parallel architectures [24], and taking advantage of the large amount of available data.

There is a growing number of studies related to the analysis of transposable elements with Machine Learning (ML) and DL. In 2021, a Python framework specialized for applying DL in genomics was published [25, 26]. In addition, DL architectures such as TERL [27], DeepTE [23], and Nakano [28] have been proposed for classification tasks, while Inpactor2 [22] integrated several neural networks for the LTR-RT detection and classification. Several ML-based tools have also been released, such as ClassifyTE [29], Transposon Ultimate [30], and Frontier [31] (for a more complete list see Table 1). Nevertheless, most of the ML and DL approaches used for TE analysis focus their core ML/DL algorithms on a single specific task (detection or classification). Thus, the other steps required to generate a complete annotation are performed by traditional (bioinformatics) approaches. Therefore, an architecture that can do both task at once can be useful for the run-times reduction.

**Table 1:** Tools and approaches that used ML or DL approaches to analyze TEs. TIR-Learner uses neural network, k-nearest neighbors, random forest, and Adaboost for the ensemble method, while ClassifyTE uses k-nearest neighbors, extra trees, random forest, support vector machine, AdaBoost, logistic regression, Gradient Boosting Classifiers and XGBoost Classifier for the stacking method. Abbreviations: RFSB: Random forest selective binary classifier, C: Classification, D: detection, A: annotation, CL: curation of TE libraries, NI: novel insertions, TU: TransposonUltimate.

Model name	Input	Output	type of model	Year	Task	TE type	Reference
TEclass	TE sequences	Plane text	SVM	2009	C	All classes	[32]
Nakano	TE sequences	Class predictions (and probabilities)	FNN	2018	C	All classes	[28]
TE-Learner	TE sequences	Plane text	RF	2018	C	LTR-RTs	[33]
TIR-Learner	Genomic assembly	Library in fasta; Annotation in GFF	Ensemble model.	2019	D, A	TIR	[34]
TERL	Any sequence	Class predictions (and probabilities)	2D CNN	2020	C	All classes	[35]
DeepTE	TE sequences	Class predictions (and probabilities)	1D CNN	2020	C	All classes	[36]
Frontier	Short reads	Plane text	2D CNN	2021	NI	All classes	[37]
ClassifyTE	TE sequences	Plane text	Stacking methods	2021	C	All classes	[38]
SENMAP	TE sequences	Class predictions (and probabilities)	2D CNN	2021	CU	LTR-RTs	[39]
Inpactor2	Genomic assembly	Library in fasta; Predictions in tab; Annotation in GFF	2 CNN, 2 FNN	2022	D, CL, C, A	LTR-RTs	[22]
TU “RFSB”	TE sequences	Class predictions (and probabilities)	RFSB	2022	C	All classes	[40]

In the computer vision scientific discipline, combined detection-classification strategies are commonly used, such as detecting regions of interest in images (called object detection) [41, 42]. In these approaches, it is possible to detect where the object is located in the images and assign it a classification. One of the most used neural network type in object detection is convolutional neural networks (CNN), in particular the architecture called YOLO (“You Only Look Once”) [43]. The principle of this network is to detect all possible objects present inside the image giving a classification to each of them, in a single execution [44]. Following YOLO’s approach, the CNN could be used to detect genomic objects in a whole genome sequence, integrating the detection task (i.e. finding the coordinates of a genomic feature such as a TE) with the classification (assigning a class to the feature such as lineages/families or superfamilies), reducing the number of individual steps, run times and complexity of TE analysis in large genomes [24]. This approach can be useful for all genomic objects (genomic regions of interest) such as coding and non-coding genes, or smaller sequences such as protein domains.

Here, we present a novel DL strategy, called YORO (“You Only Read Once”), for the detection and classification (lineage/family and type) of LTR-RT internal domains, which is integrated into a pipeline. This strategy provides the basis for future research where complete LTR-retrotransposons could be annotated and classified using a purely DL approach. The methodology adopted and proposed here includes a description of the input data, label creation, training data set generation, architecture design, training configuration, post-processing, and visualization of the model results. Finally, the metrics used to evaluate the performance of YORO are described.

## Results and discussion

### 0.1 The genomic object detection approach

In order to integrate both detection and classification into a single task, an approach based on genomic object detection was designed. This approach is based on the existing concept in computer vision, according to which a neural network is able to detect subregions of interest within an image and subsequently classify them according to its training database. The DL model that applies this approach (called YORO) is based on convolutional neural networks and residual networks that facilitate learning and continuous decrementing of the loss function. YORO receives genomic sequences of 50,000 bp length and divides them into 100 bp sections and predicts for each section the presence/absence of domains, start position, domain length, to which lineage/family it belongs and what type of domain it is (GAG, AP, INT, RT, RH, or ENV). The YORO output is processed with the non-max suppression method (NMS) to eliminate redundancy and at the end delivers a tabular text file with the information on predicted domains.

### 0.2 Training data set and model training

To train the CNN, a synthetic data set was created from real information of LTR-RT. Basically, a bunch of LTR-RT taken from InpactorDB [15] was randomly placed inside an entire DNA sequence with a fixed length of 50, 000 bp. The nucleotides filling the space between one LTR-RT and another corresponded to sequences that are known to not contain LTR-RT (negative data set taken from [45] DOI: 10.5281/zenodo.4543904, See Methodology section). After the synthetic creation of DNA sequences, they were transformed into a one-hot 2D representation and they were used as features for training the CNN. Each 50, 000 bp sequence was divided into 100 bp sections and for each section we created a set of 22 labels representing presence or absence of a domain in the 100 bp section, starting position, length, lineage/family, and type of domain. We also added some genomic sequences having only negative sequences (i.e. not containing any LTR-RT sequence) to simulate regions devoid of LTR-RTs.

### 0.3 Performance metrics

**Fig 2:**
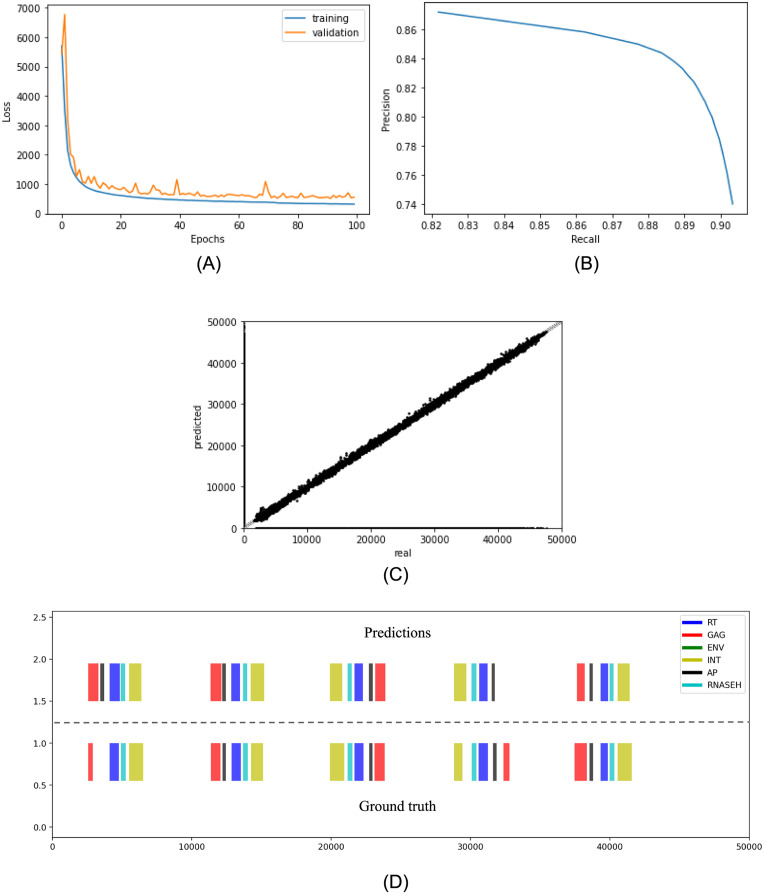
Performance of YORO in detecting internal domains of LTR retrotransposons using the Genomic Object Detection approach. (A) Loss function during model training. Parameters used: Adam algorithm, learning rate of 0.001, batch size of 128, number of epochs 100, no droputs, data split: training (80%), validation (10%), testing (10%). (B) Precision-Recall curve with TP (True Positive), TN (True Negative), FP (False Positive) and FN (False Negative) defined on a nucleotide basis. Only domain detection is considered, regardless of its classification. (C) Parity plot for the positions of the beginning of the domains. (D) Visualization of the domains in the 50,000 bp window (X-axis). The upper part corresponds to the predictions done by YORO. The lower part corresponds to the actual label. AP: Asparic Protease (black); GAG: Capside protein (red), ENV: Enveloppe (green), INT: Integrase, RT: Reverse Transcriptase (blue), RNAseH: Ribonuclease H (light blue).

While training the developed residual CNN, the loss function displayed no overfitting problems since the difference between the validation and training curve was less than the difference between training and ground truth (Fig 2A). Therefore, bias should be the first problem that should be addressed in future research. The raw predictions generated by YORO were filtered in two ways: (i) presence probability > 0.1 and (ii) duplicate reduction by NMS (non-max suppression, See Methodology section), a procedure used in object detection for images to remove any duplicates. The presence probability threshold was selected as the threshold that outputs the largest product between precision and recall. To do so, a precision-recall curve was constructed (Fig 2B). The largest product was found at a threshold of 0.8, where precision and recall were 0.8435 and 0.8837. The threshold-processed predictions were compared with the ground truth using a parity plot (Fig 2C) and the visualization procedure (Fig 2D).

The parity plot compared the initial position of each domain within the 50, 000 nucleotide window for predicted and actual labels. Dots on the vertical axis corresponded to false positives (FP) domains, while those on the horizontal axis corresponded to false negatives (FN). In this comparison, each domain was compared independently of its classification. The coefficient of determination computed for this set of points yields a value of *R*^2^ = 0.7083. Although most of the points are remarkably well distributed around the diagonal, the FP and FN prevented the distribution from reaching a high coefficient of determination. This coefficient is indeed an evaluation of the force between the two variables determined. In a perfect scenario the *R*^2^ would be 1, indicating that both variables are actually the same. The visualizations of the predicted domains against the real data provided further insight (Fig 2D and Fig S2 in S1 File).

The upper part in Fig 2D corresponds to domains predicted by YORO and the lower part to the real labels. Some domains predicted by the model were not present in the real label, and at the opposite some domains in the real label were not present in the predicted result. This situation was repeated in most of the samples in the data set. Nevertheless, some of the domains classified as false positives were located in positions supported by real LTR-RT. For example, in Fig 2D, the first group of domains (left to right) contained an extra domain classified as AP (Aspartic Protease) by the model but was not present in the real label. The AP domains were expected to be present between the GAG and the INT domains in Ty1/copia elements or between the GAG and the RT domains in Ty3/gypsy elements [8]. This could be due to imperfect annotation of the domains when the data set was created.

The classification of each domain by YORO was evaluated by using a COCO evaluator and the mean average precision (COCO mAP@0.5). Regarding the classification based on the protein type, the mAP calculations were summarized in Table 2. It was clear that the number of ENV domains produced a class imbalance problem, thus ENV domains were probably not well predicted by YORO. However, the RT, INT and RH domains obtained the highest mAP as expected because their relative high conservation between LTR-RT elements in plant genomes [11]. In contrast and despite the high number of samples in the data set, GAG exhibited the lowest performance. This could be attributed to the high levels of sequence divergence, absence of highly conserved sites, and the presence of stop codon and frame shift mutations in the coding region of many elements [11, 46]. Regarding lineage classification, the mAP results were displayed in Table 3. The IKEROS lineage had very few samples in the training data set, so YORO did not correctly learn how to identify this lineage/family.

**Table 2:** COCO mAP@0.5 calculations for domain classification.

Domain class	COCO mAP@0.5 (%)	class contribution (%)
RT	91.6	20.7
GAG	70.7	19.1
ENV	40.6	0.4
INT	85.4	20.7
AP	85.3	18.7
RH	90.3	20.4
mean	77.35	-

**Table 3:** COCO mAP@0.5 calculations for lineage classification of the domains.

LTR-RT Lineage class	COCO mAP@0.5 (%)	class contribution (%)
ALE-RETROFIT	84.6	18.9
ANGELA	85.8	5.6
ATHILA	79.5	4.5
BIANCA	57.4	2.0
CRM	84.6	3.6
DEL-TEKAY	85.4	14.9
GALADRIEL	74.7	1.5
IKEROS	0	0.1
ORYCO-IVANA	84.7	5.2
REINA	76.9	5.1
SIRE	82.0	5.5
TAT	86.6	22.8
TORK-TAR	81.9	10.3
mean	74.20	-

In general terms, the mAP values for the different categories indicated that the model performed well in the classification task when compared to different versions of YOLO in [47] where the mAP ranged from 63.4 to 78.6.

**Fig 3:**
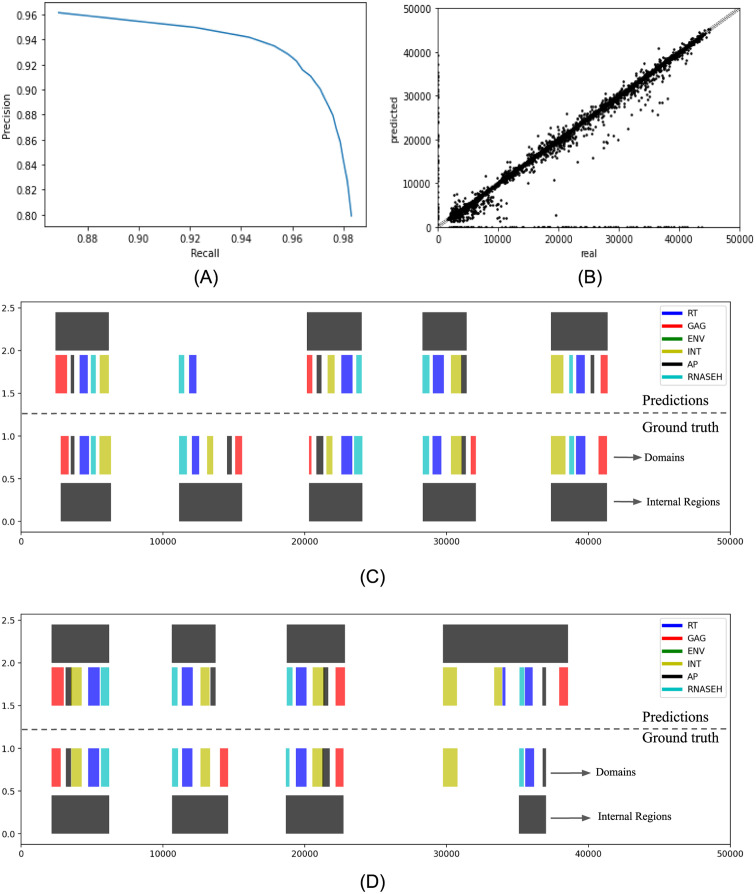
YORO’s performance in detecting LTR-retrotransposon’s internal part grouped by at least three domains with a maximum distance of 3, 000 bp, according the Genomic Object Detection approach. (A) Precision-Recall curve with TP (True Positive), TN (True Negative), FP (False Positive) and FN (False Negative) defined on a nucleotide basis. Clusters with a minimum of three domains and a maximum separation of 3, 000 bp. (B) Parity plot for the positions of the beginning of the clusters with a minimum of three domains and a maximum separation of 3, 000 bp. (C) Visualization of the clusters with a minimum of three domains and a maximum separation of 3, 000 bp, in the 50, 000-bp window. The top section corresponds to the predictions by YORO. The bottom section corresponds to the real label. There is a false negative. (D) Visualization of the clusters with a minimum of three domains and a maximum separation of 3, 000 bp, in the 50, 000-bp window. The upper section corresponds to the predictions by YORO. The lower section corresponds to the real label.

Since the model predicted domains (FP and TP) in the regions where an LTR-RT is expected to be present, the precision-recall curve is computed again but this time clustering groups with a minimum of three domains and separated by a maximum distance of 3, 000 bp from each other. The resulting curve (Fig 3A) exhibited a great improvement in both metrics precision and recall. The maximum product lied in a probability threshold presence of 0.8 where precision and recall were 0.9350 and 0.9527.

The internal regions considered as False Positive and False Negative were less numerous since the dots located on the vertical and horizontal axis of the parity plot (Fig 3B) were less numerous to the previous diagram. Additionally, the determination coefficient for the distribution of the dots in the parity plot was *R*^2^ = 0.9379, which was higher than the previously computed value.

Visualizing some random samples (Fig 3C and 3D) gave some insights about the improvements obtained by taking clusters with a minimum of three domains and showed some details that still need to be improved. For example, in Fig 3C, if the model only predicted only one or two domains in which an LTR-RT is present, the cluster could not be identified and a false negative was generated. Fig 3D illustrated an issue associated with the clustering approach: domains in the actual labels were separated by a variable number of nucleotides, even greater than 3, 000 bp. This fact leads to situations where the prediction was clustered appropriately but the real label was not, as confirmed by the last cluster in Fig 3D.

From the Fig 3D, a hypothesis was put forward be proposed: it was possible that YORO performed even better than the metrics indicate. With this in mind, a new performance analysis was sketched by evaluating YORO in the genome of *Oryza Sativa* ssp. *indica* and comparing the predictions against the annotation available in [48]. The results of this comparison are summarized in Table 4. In all the cases the metrics are computed on a nucleotide basis.

**Table 4:** Precision and recall for YORO predictions on the *Oryza Sativa* ssp. *indica* genome versus its publicly available annotation [48]. GT: Ground Truth. P: process applied.

Case	Min. dom.	Precision	Recall	GT Description	P description
A	1	0.6574	0.2518	Complete LTR-RT	Clustering
B	2	0.7074	0.2440	Complete LTR-RT	Clustering
C	3	0.7888	0.2327	Complete LTR-RT	Clustering
D	4	0.8496	0.2179	Complete LTR-RT	Clustering
E	5	0.8806	0.1892	Complete LTR-RT	Clustering
F	5	0.9507	0.1000	Complete TE-RT	Clustering
G	1	0.7442	0.4206	Complete LTR-RT	Clustering + LTRharvest
H	2	0.7833	0.4129	Complete LTR-RT	Clustering + LTRharvest
I	3	0.8484	0.3862	Complete LTR-RT	Clustering + LTRharvest
J	4	0.8952	0.3594	Complete LTR-RT	Clustering + LTRharvest
K	5	0.9177	0.3070	Complete LTR-RT	Clustering + LTRharvest
L	1	0.8047	0.3675	Complete LTR-RT	Clustering + LTRharvest + filter
M	2	0.9455	0.3030	Complete LTR-RT	Clustering + LTRharvest + filter
N	3	0.9680	0.2829	Complete LTR-RT	Clustering + LTRharvest + filter
O	4	0.9819	0.2655	Complete LTR-RT	Clustering + LTRharvest + filter
P	5	0.9886	0.2266	Complete LTR-RT	Clustering + LTRharvest + filter
Q	1	0.6004	0.4799	Internal region	Clustering
R	2	0.6466	0.4652	Internal region	Clustering
S	3	0.7217	0.4441	Internal region	Clustering
T	4	0.7782	0.4163	Internal region	Clustering
U	5	0.8032	0.3599	Internal region	Clustering
V	5	0.9600	0.3900	Complete LTR-RT	RepeatMasker (case P) + div 5
W	5	0.8000	0.8100	Complete LTR-RT	RepeatMasker (case P) + div 20

In cases A through E in the Table 4, domains separated by a maximum distance of 4, 000 bp were clustered in a single region. Each case was differentiated by the minimum number of domains required for clustering (column “Min. dom.”). In this sense, case A formed clusters with a minimum of one domain, while case E formed clusters with a minimum of five domains. The precision ranged from 0.6574 to 0.8806, indicating that the detection of multiple domains in a nearby region was an indication of the presence of a LTR-RT. Nevertheless, recall decreased as the number of domains needed to form a cluster increased, which implied a loss of predictions as clustering become more stringent. Furthermore, recall was low for all the cases, as the flank of the LTRs were not predicted and contributed to the false negatives term when computing the metrics. In cases A-E, the predictions were compared against the annotation with the tag “LTR”. The case F is similar to E but the predictions were compared to the complete annotation, independent of the TE classification. In this situation, the precision reached 0.9507, indicating that the model was predicting internal regions not only for LTR-RT but also for other TEs. This may due to the fact that similar protein domains can be found in TE from other orders such as GAG, RH and RT domains in LINE elements, GAG, AP, RT, and RH in DIRs elements and RT in PLE elements or to imprecise annotation in the public version of the annotation [8].

The internal regions predicted in cases A-E were then extended 8, 000 bp to the left and right. These segments were passed to LTRharvest to include long terminal repeat regions detection. The metrics for the resulting predictions were comprised in cases G through K. There was an overall improvement in the precision and recall ranging from 0.7442 to 0.9177 and from 0.4206 to 0.3070, respectively. This meant that the use of LTRharvest helped to remove false positives.

In cases L through P, the LTRharvest tool was applied to the predictions of cases A-E to detect the LTR flanking regions and discard those internal regions that did not have LTR-flanks. There is an overall improvement in the precision and recall ranging from 0.8047 to 0.9886. This meant that the use of LTRharvest helped to remove false positives. However, the recall metric remains low in all the cases.

The annotation includes an additional feature to distinguish the internal part of an LTR-RT from the LTR flanking regions. Taking advantage of it, predictions of cases A-E were now compared to the internal parts of the annotation, yielding the results in cases Q-U. The precision was overall lower than the corresponding previous cases. Such a decrease in precision could be explained by the fact that the definition of the internal region was not specified. For instance, the predictions defined the internal region between the start of the first coding domain and the end of the last coding domain. However, the annotation could defined the internal region from where the 5’ LTR-flank ends and where the 3’ LTR-flank ends.

Taking the predictions with the best precision value (case P) as a TE library, RepeatMasker was applied over the genome with the following parameters “-s -pa 10 -nolow -cutoff 225 -gff -no_is -norna”. The results were evaluated for two values of -div: 5 and 20. The final metrics with this approach corresponded to cases V and W. An allowed divergence of 20 decreased the false negatives but also increased the false positives, as can be deduced from precision (0.80) and recall (0.81). A more stringent divergence value of 5 kept false positives low (yielding a high precision, 0.96) but did not decrease significantly false negatives (yielding a low recall, 0.39).

### 0.4 Execution times in different genomes

One of the main bottlenecks in genomics today is the time required to analyze the enormous amount of data produced by the new sequencing technologies [49, 50]. We therefore wanted to test our Genomic Object Detection approach and the YORO architecture on a large-scale study. To this end, 91 plant species genomes were randomly selected from the 300 genomes data set proposed by [12]. The selected species had a size distribution between 300 Mbp and 3.2 Gbp and in total they summed 240 Gbp. Our approach took 10.18 hours to run with all the genomes (Fig S3 in S1 File) on a server with 62 cores and an NVIDIA A100 GPU with 20 GB of memory, a considerably short time compared with conventional *de novo* Transposable Elements detection tools.

### 0.5 Comparison against conventional tools

BLAST (Basic Local Alignment Search Tool) is still very popular and is used to identify protein domains in nucleotide sequences (BLASTx algorithm). Fig S4 in S1 File shows a comparison of the number of domains detected by YORO and BLAST in 17 plant genomes (Table S1 in S1 File). The difference between the two methods was consistently positive across the graph, indicating that YORO detected more domains than BLASTx. In addition, a positive correlation between the difference and genome size was observed, with an *R*^2^ value of 0.94 (Fig S4 in S1 File). These findings suggested that the BLASTx’s ability to detect domains was limited by the availability of homologous sequences in the reference database. Consequently, domains that are specific to some species may remain undetected. This problem is more pronounced in larger genomes, as the proportion of undetected domains increased relative to the number of detected domains.

On the contrary, observations demonstrated that YORO’s ability to detect domains was directly correlated with genome size. This implied that the algorithm was not only limited to recognizing the domains it has been was trained on, but also capable of identifying other domains sharing similar structural characteristics with the training set.

**Fig 4:**
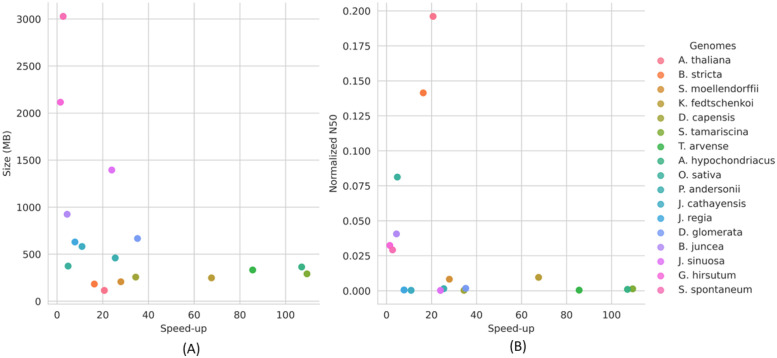
Relationship between speed-up and assembly variables analyzed by YORO and BLAST: (A) speed-up vs genomes size (sample: 17 plant genomes), and (B) speed-up and normalized N50 value.

Fig 4 shows the correlation between genome size and the speed-up of YORO compared to BLASTx in terms of execution time. In all cases, YORO outperformed BLASTx, exhibiting up to 109 times faster performance. Notably, the speed-up was non-linear and tended to decrease with increasing genome size. To further investigate this relationship, we analyzed other variables such as assembly quality, as reflected by the normalized N50 value, and speed-up, as shown in Fig 4B. In this case, no correlation was found between these variables. However, we observed that the number of elements detected by YORO contributed to this behavior, especially in larger genomes, as shown in Fig 4A. In such cases, YORO took more time to perform functions such as IOU computation, Non-max suppression, and writing the output file.

### 0.6 Comparison between domain distances

**Fig 5:**
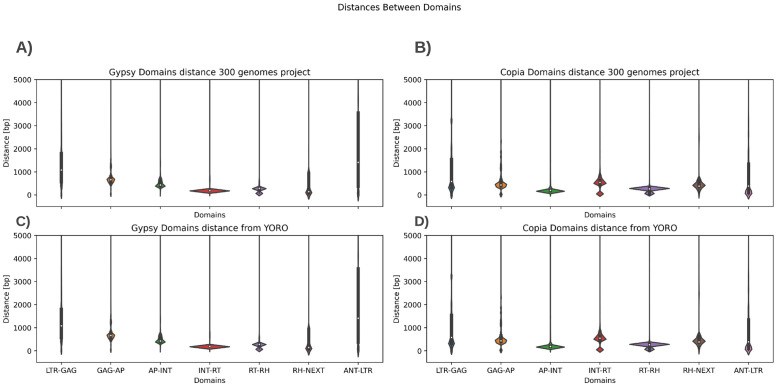
Nucleotide distances between domains reported by [12] for: (A) Ty3/gypsy and (B) Ty1/copia, and predicted by the Genomic Object Detection approach (throught YORO) for: (C) Ty3/gypsy and (D) Ty1/copia.

Having obtained positive results in precision, recall and also in execution times, we wanted to check whether the distances between predicted genomic objects were also accurate or not. To answer this question, we compared the nucleotide distances between the LTR-RT domains identified using YORO and the distances previously proposed by Zhou and co-authors using an HMM search [12]. First, the distances between domains were extracted for both data sets: the one obtained from Genomic Object Detection approach and the published genome data set [12]. To this end, the corresponding files were processed and the average distances between the different LTR-RT domains present in the genomes were determined. These distances provide valuable information about the structure of the elements and make it possible to assess the similarity between the results of the two methods. Once the distances between domains had been obtained, a series of comparative analyses were carried out to evaluate the agreement between the results of the YOLO approach and those of the reference program used in [12] (LTRharvest/LTRdigest). Descriptive analyses were conducted, including the calculation of means and standard deviations, as well as the generation of violin plots to visualize the distribution of the distances between domains in the two data sets (Fig 5).

The results obtained show that the distances between domains of the LTR-RTs identified by YORO exhibited a similar distribution to reported in the 300 genomes project [12]. Although some differences in the average distances between domains were observed, these were not statistically significant, suggesting that the Genomic Object Detection approach was capable of providing results comparable to those of the commonly used programs in terms of detecting and characterizing LTR-RTs (Tables 5 and 6 to see the mean distance between all domains from the used data sets).

**Table 5:** Average distance between domains for the Ty1/copia superfamily as observed in the analysis of 300 plant genomes [12] and in the YORO prediction.

Distance between	Observed Mean distance*** (bp)	YORO Mean distance (bp)
5’-LTR-GAG	1084.14	1823.35
GAG-AP	633.76	715.14
AP-INT	268.81	241.36
INT-RT	544.63	605.65
RT-RH	244.31	312.85
RH-NEXT*	703.83	625.78
PRE-LTR-3’**	966.62	1235.69

* Distance between RH domain and the next domain of the element.

** Distance from any domain preceding the 3’ LTR domain.

*** Observed distances found by [12]

**Table 6:** Average distance between domains for the Ty3/gypsy superfamily as observed in the analysis of 300 plant genomes [12] and in the YORO prediction.

Distance between	Observed Mean distance*** (bp)	YORO Mean distance (bp)
5’-LTR-GAG	1379.26	1563.69
GAG-AP	760.8	680.61
AP-RT	552.2	620.31
RT-RH	242.27	318.15
RH-INT	277.92	198.26
INT-NEXT*	694.03	801.65
PRE-LTR-3’**	2088.2	1902.18

* Distance between the INT domain and the next domain of the element.

** Distance from any domain preceding the 3’ LTR domain.

*** Observed distances found by [12]

## Conclusion

The YORO approach can be used successfully to detect genomic objects. It detects individual LTR-RT domains with a precision of 0.8435 and a corresponding recall of 0.8837. It classifies individual domains with an mAP of 77.35% for protein domain type and 74.2% for lineage/family classification. It detects the inner regions of LTR-RTs with an accuracy of 0.9350 and a corresponding recall of 0.9527. YORO predicts the position of LTR-RT internal regions with a correlation coefficient of 0.9379 for a parity plot against real labels, and predicts LTR-RT internal regions for the *Oryza Sativa* ssp. indica genome with a precision of 0.9507. By filtering the YORO output with LTRharvest, the accuracy increases to a value of 0.9877. This approach demonstrated low run time, analyzing 91 genomes in less than 10 hours and detecting genome-level LTR-RT domains up to 109 times faster than the BLAST algorithm. Finally, the distances between domains predicted by YORO were in line with the observed data sets (LTR-RT annotation of 300 plant genomes). In this sense, as a proof of concept YORO provides a framework for future improvements in the detection and annotation of individual LTR-RT domains to increase the sensitivity and accuracy of the full annotation of LTR-RT elements. Further effort will be required to include a detection/prediction step for LTR-RT extremities, without using external tools which could slow down YORO, and which could reinforce the quality of its predictions. YORO could be integrated as a tool for annotation, classification or re-classification of LTR-RT elements in a similar way to tools based on HMM profiles, and also offers the possibility of including more genomic objects such as other classes of elements (transposons, helitrons), or coding regions. In the future, we aim to develop finalized tools for the community capable of detecting and classifying TEs based on this architecture.

## Methodology

### 0.7 Inputs and outputs

DNA sequences are composed of four nucleotide: adenine (A), cytosine (C), guanine (G), and thymine (T). Depending on the genome assembly quality, other letters are used to represent a nucleotide that is not fully characterized in one of the four categories (e.g. “N” represents any nucleotide). Since a CNN relies on numerical inputs, a mathematical transformation is required for the DNA sequences. In previous research [27], a one-hot encoding strategy is utilized to transform DNA sequences into a mathematical representation ready to be fed into a CNN. Here, a slight modification of such one-hot encoding is used. A matrix of dimensions 4*xN* is built from a DNA sequence, where *N* is the sequence length and 4 corresponds to the four nucleotide types in the following order: A, C, G, T (Fig S1 in S1 File). Any other letter is represented by a column of zero.

The purpose of the CNN model is to detect and classify internal protein coding regions of LTR-RT, namely, group antigens (GAG), aspartic proteinase (AP), integrase (INT), reverse transcriptase (RT), RNase H (RH), and envelope protein (ENV) (Fig 1). Hence, the information related to the domain position, size, type (GAG, AP, etc), and classification (in lineages/families) is encoded in labels for model training.

**Fig 6:**
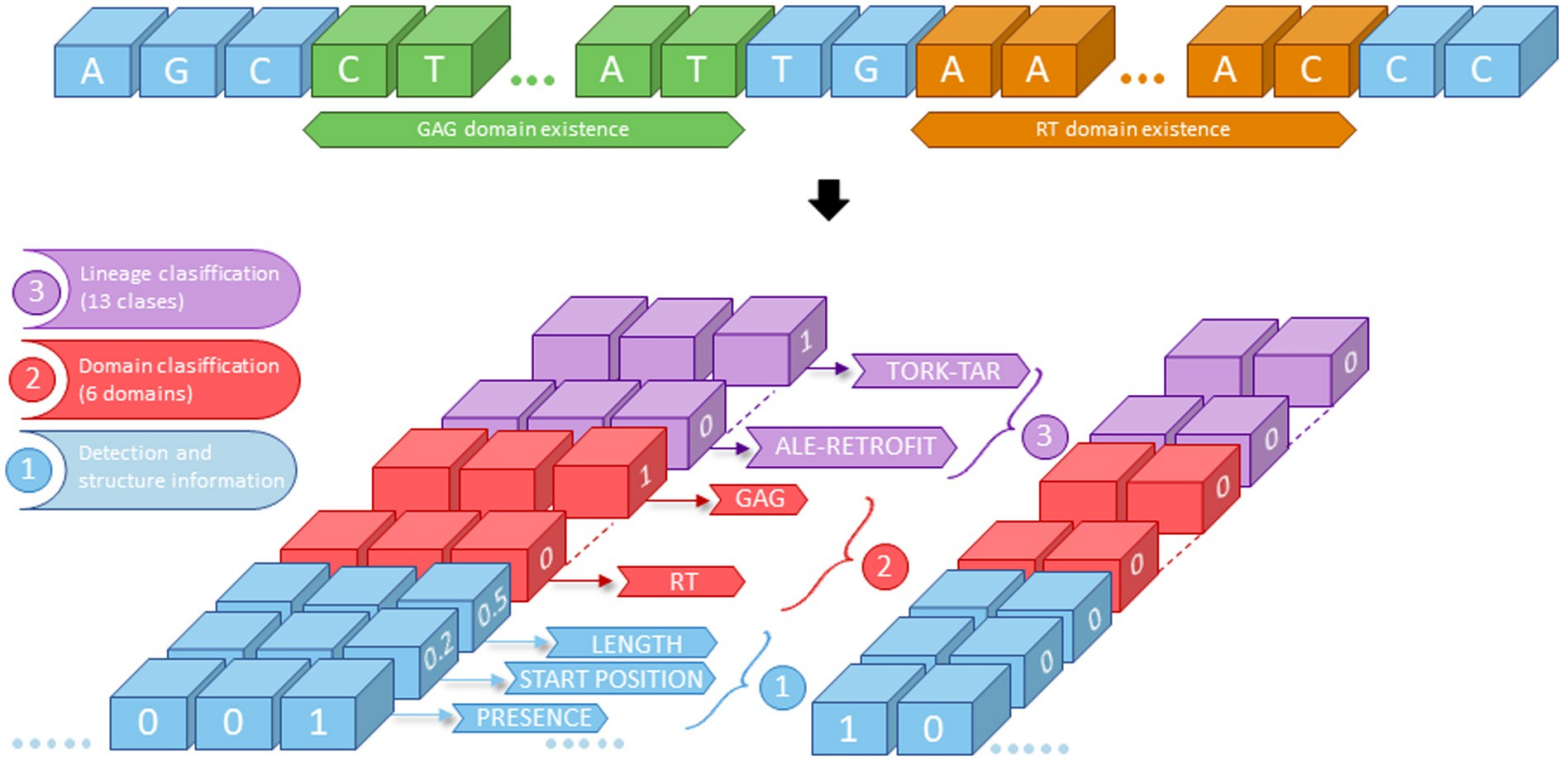
Structure of the labels encoding the domain information of the LTR-RT domains. The initial nucleotide sequence (upper section) is divided into 100 bp sections each one with 22 labels (lower section). Those labels represent the following: (1) Detection and structure information: Presence of a domain, starting position of the domain in the 100 bp section and length; (2) Domain classification: to which domain it is related (in one-hot coding, e.g. GAG = 1,0,0,0,0,0); (3) Lineage classification: to which lineage the domain is related (in one-hot coding, e.g. Tork = 1,0,0,0,0,0,0,0,0,0,0,0,0). Both the domain start position and the length are normalized to be values between 0 and 1. Following this approach, a neural network can learn how to do three different task at once (domain detection, domain classification, and lineage classification).

In YOLO’s original paper [43], the image to be analyzed is split into a 7*x*7 grid, each with its own label containing information about the presence or absence of an object, its size, and its classification. Similarly, the DNA sequence is divided into groups of 100 nucleotides and each group has its own label with 22 coordinates (Fig 6). The first number corresponds to the presence of a domain (one for presence and zero for absence). The second number represents the relative position of the beginning of the domain within the 100 bp section. It is a number between zero and one. The third coordinate is the domain size relative to the maximum length of each domain class. The next six coordinates correspond to the domain type into one of the six groups. And the last 13 coordinates indicate the lineage classification of the domain. The lineages that were considered in this research are (1) *Ale/Retrofit* (2) *Angela* (3) *Athila* (4) *Bianca* (5) *Crm* (6) *Del/Tekay* (7) *Galadriel* (8) *Ikeros* (9) *Oryco/IVANA* (10) *Reina* (11) *Sire* (12) *Tat* (13) *Tork/Tar*.

### 0.8 Data set generation

The inputs data sets were created using the following steps:

1. Create a synthetic DNA sequence of 50, 000 bp by concatenating sequences known to not include any LTR-RT (i.e coding sequences, different types of RNA like mRNA, tRNA, non-coding RNA, and other types of TEs such as TEs Class II) from [45] DOI: 10.5281/zenodo.4543904. These sequences are called “negative background”.
2. Generate random numbers between 3, 000 and 4, 000 that designate the number of nucleotides remaining between the TEs.
3. Randomly select TEs from InpactorDB [51].
4. Replacement of the corresponding section in the negative background with the selected TEs, considering the number of nucleotides remaining between the TEs. Note: The number of TEs inserted in the negative background depends on their lengths. The position, length, class and lineage of the protein-coding domains within each TEs are stored for the labels data set creation.
5. Repeat steps one to four until all TEs in InpactorDB are used.
6. Transform all DNA sequences generated by this procedure into one-hot encoding representation.

After performing the mentioned steps, a tensor is created with dimensions *Mx*4*x*50, 000 where *M* is the number of samples. In addition, the label data sets are created simultaneously with the input data sets:

1. Create a tensor of zeros with dimensions *Mx*1*x*500*x*22
2. Divide the domain position by 100 and take the quotient (an integer) and the remainder. Note: The quotient represents the *i-th* 100-nucleotide box where the beginning of the domain is found.
3. Divide the remainder by 100 and leave the quotient with at least two decimals. Note: This number represents the relative position of the domain inside the 100-nucleotide box.
4. Divide the domain length by the maximum size of the same domain type reported in InpactorDB with full decimals. Note: this number indicates the normalized domain size.
5. Iterate over the tensor from step one and record in the last dimension with 22 elements the presence/absence (index 0), position (index 1), length (index 2), type (index 3 − 8), and lineage/family classification (index 9 − 21).

### 0.9 Architecture

The proposed architecture is based on a CNN with a residual learning strategy. In [52], image recognition models are significantly improved by implementing a deep residual learning framework in which direct access connections that perform identity mapping are added to the architecture. These shortcuts skip one or more layers. Such stacked layers are the building blocks of a residual network. Some of the benefits of such a strategy are:

- It addresses the training accuracy degradation problem generated by the high number of layers in deep networks.
- It eases the optimization of residual mapping
- The entire network can still be trained end to end with back-propagation and implemented with common libraries.
- It is expected to be also applicable in other fields besides computer vision.

**Fig 7:**
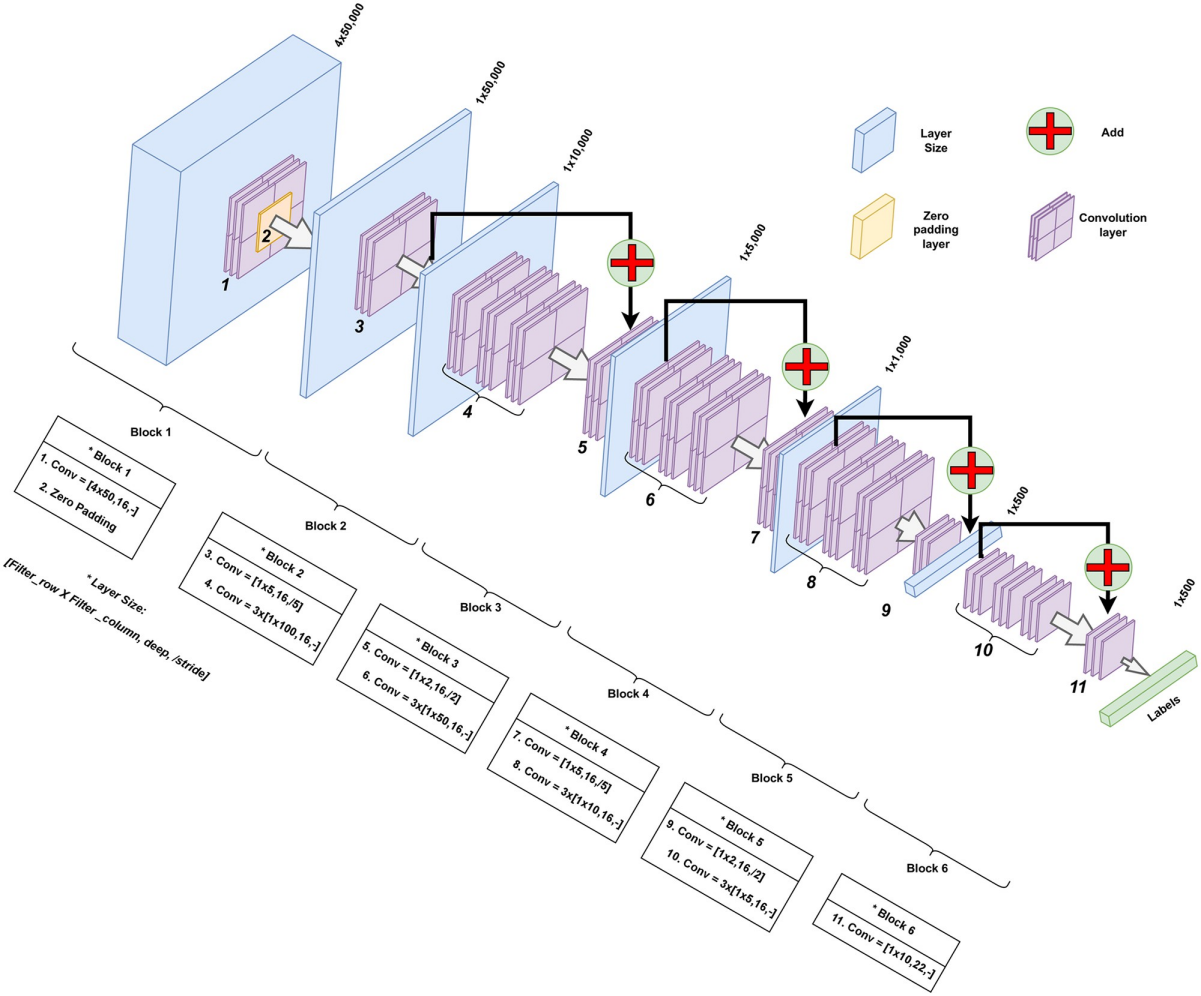
Neural network architecture of YORO.

The architecture is basically composed of six blocks (Fig 7). The first one processes the input with a filter of 4*x*50*x*16 where 4 corresponds to the four rows of the one-hot encoding, and 50 and 16 were set after experimentation with the network. The following four stacks are residual blocks that reduce the size by a factor of five, two, five, and two, respectively. Each block has its own shortcut connection. The last block corresponds to a layer needed to adjust the output of the model as required by the label formatting.

### 0.10 Model training

For model training, a loss function is adapted from [43] (Eq 1). It calculates the sum-squared error for presence probability (*p*), position(*x*), size (*w*), and classification (*c*). Considering that most of all grid cells do not contain protein-coding domains, it is necessary to weigh the loss function, by giving more relevance to the cells where there is a domain. This is accomplished by using λ_*obj*_ and λ_*nonobj*_. According to previous experiments, these values yield good results depending on the number of domains inside the window of 50, 000 nucleotides. In this research such weights are set to λ_*obj*_ = 1 and λ_*nonobj*_ = 0.06. In Eq 1, $1_{i}^{obj}$ denotes if a protein-coding domain appears in cell *i*.

$$\begin{array}{c} \begin{array}{cc} & \lambda_{obj}\sum_{i=0}^{S}1_{i}^{obj}[{\left(p_{i}-{\hat{p}}_{i}\right)}^{2}+{\left(x_{i}-{\hat{x}}_{i}\right)}^{2}+{\left(w_{i}+{\hat{w}}_{i}\right)}^{2}]\\ & +\lambda_{obj}\sum_{i=0}^{S}1_{i}^{obj}\sum_{j=0}^{N_{C}}[{\left(c_{i,j}-{\hat{c}}_{i,j}\right)}^{2}]\\ & +\lambda_{nonobj}\sum_{i=0}^{S}1_{i}^{nonobj}[{\left(p_{i}-{\hat{p}}_{i}\right)}^{2}] \end{array} \end{array}$$ (1)

During model training, the following parameters were used:

- Weights initialization strategy: Xavier normal initializer (“glorot_normal” option in TensorFlow2)
- Optimizer: Adam algorithm
- Learning rate: 0.001
- Batch size: 128
- Epochs: 100
- No dropouts are used
- Data partition for training, validation, and testing: 80%, 10%, and 10%, respectively.

### 0.11 Post-processing: Non-max suppression

After the training stage, the model is evaluated on the test partition of the data set. The predictions generated by the network must be filtered in two steps. The first step filters by comparing the probability of presence to a threshold. If the probability of presence is greater than the threshold (> 0.1), we say that there is an object in the grid cell. Nevertheless, some of the remaining predictions may point to the same protein-coding domain. To overcome this multiplicity of predictions for the same domain, the second step filters the predictions by leaving only one. To this end, non-max suppression (NMS) is an usual procedure used in object detection for images. Herein, the non-max suppression strategy is utilized with an intersection over union (IoU) threshold of 0.1. In images, it is normal to locate different objects in the same region since the image is transforming a 3D space down to two dimensions. Therefore, two objects in an image can be identified in the same grid cell, one behind the other. This fact led modern object detection systems in images to use the concept of bounding boxes. however, in DNA sequences, there is only one dimension, so there is not such an overlapping concept for genomic objects. The IoU threshold should be set to zero, but it was decided to keep a small margin of error for predictions.

### 0.12 Output visualization

During data set creation and model testing, it is imperative to implement a strategy to visualize the actual labels against the predicted labels, which facilitates the identification of potential deficiencies into which the model may fall. Consequently, each domain is represented as a horizontal line of a specific color in a window of 50, 000 units in length representing the number of nucleotides in each sample in the data set.

### 0.13 Performance metrics

The performance metrics used to evaluate YORO include precision, recall, accuracy, F1-score, precision-recall curve, parity plot for predicted domain positions, and mean average precision (mAP).

The computation of recall, accuracy, precision, and F1-score relies on the definition of true positives (TP), true negatives (TN), false positives (FP), and false negatives (FN). According to [22], these quantities are defined on a nucleotide basis for genomics-related deep learning models. In this research, the same definitions have been used. However, it is important to clarify that predictions are considered true positives as long as the domain prediction coincides with any other domain in the ground truth label, regardless of the domain classification. In this sense, if the network predicts a domain in the same position as the label but they are of different type, the prediction is still considered a true positive. At this stage of the analysis, only the domains are being compared without considering their types in order to evaluate the capacity of the network to predict the position and size of internal domains.

On the other hand, the classification of each predicted domain is evaluated through the mAP. The mAP calculation is based on COCO mAP@0.5 and is performed for the domain categories (AP, INT, ENV, RT, GAG, and RH) as well as lineages/families.

Precision, recall, accuracy, and F1-score were analyzed from two perspectives: (A) individual domains and (B) clusters with a minimum of three contiguous domains separated by no more than 3, 000 nucleotides. The second approach aims to analyze whether YORO can be used to detect the entire internal region of LTR-RT in a genome, that is, excluding the flanks of LTR.

A parity plot is also used to measure the performance of the model predictions by comparing the position of the start of each domain for the predicted and real labels. Additionally, another parity plot is constructed by comparing the start of each internal region.

Although the data set is somewhat synthetic, the trained model is also tested on the genome of *Oryza sativa* ssp. indica, since its annotation is well curated and available in [48]. The precision and recall are computed on a nucleotide basis for the internal regions detected by YORO.

YORO is intended to be improved by the research community to be able to annotate all type of transposable elements contained in a given genome. A proof of concept to illustrate this point, is to apply LTRharvest [53] on the internal regions detected by YORO and obtain the complete LTR-RT. This result is compared with the annotation from [48] in terms of precision and recall. The parameters used by LTRharvest are: -seqids yes -maxlenltr 3000 -similar 80.

### 0.14 Execution times in several genomes

In order to evaluate the performance of the Genomic Object Detection approach proposed in this study, a performance test was conducted by running the pipeline with 100 different genomes from the 300 plant genomes previously annotated for LTR-RT [12], with genome sizes ranging from 300*Mbp* to 1.4*Gbp*. This allowed us to evaluate the model performance in terms of run-time with complete and large genomes, and to obtain information on the speed of the LTR-RT’s internal domain detection-classification task.

### 0.15 Comparison against conventional tools

The performance of YORO was compared against BLAST (Basic Local Alignment Search Tool) [54], a conventional approach used for the detection and classification of LTR-RT domains. For this end, both approaches were run on 17 genomes (Table S1 in S1 File), randomly selected from the genomes used in [12]. The variables of execution time, speed-up (ratio of BLAST and YORO times) and number of domains detected by each of the genomes were measured. Genome-specific variables such as size and N50 values were also measured. In the case of the N50 values, it was normalized in relation to the genome size in base pairs (bp). BLASTx was used by applying the parameters reported in [55], with a evalue of 1e-4 and as this program uses a homology-based mechanism for detection, a well-curated reference database is crucial for accurate analysis. For this end, the combined domains found in the REXdb [11] and the GyDB [56] databases were used. YORO was executed using a threshold of 0.8 with the remaining parameters set to their default values. The computational resources used for this analysis included 32 CPU cores, 250 GB of RAM, and one NVIDIA A100-PC GPU with 20 GB of RAM.

### 0.16 Comparison of distances between domains

The performance of YORO on domain detection was evaluated, comparing it to an annotation of the same domains performed by [12] in plant genomes using an approach based on HMM profiles. In this experiment, we compared the output file of YORO (a tabular file of domain position) with the annotation performed by [12] (a GFF file). We pre-processed all the files, calculating the distance in base pairs between each detected domain of the same element, taking into account the order of the domains for each of the LTR-RT superfamilies (Ty3/gypsy and Ty1/copia). Due to the nature of some LTR-RTs, additional domains are inserted at the end of the element before the LTR, so to calculate distances, the ANT and NEXT labels were added. NEXT refers to the distance between the specific domain and the next, whatever that may be, and ANT is the distance between the previous domain and the final LTR of the same element.

Next, we calculated the average distances for each of the tested combinations, and finally, in a violin plot, we displayed the distribution of the distances and tabulated the average distances for each of the superfamilies.

## Supporting information

S1 File File containing figures S1-S4 and Table S1. (PDF)
