# Supplementary material for: Genomic object detection: An improved approach for transposable elements detection and classification using convolutional neural networks
Source: PLoS One. 2023 Sep 21;18(9):e0291925. doi: 10.1371/journal.pone.0291925 (PMC10513252; doi:10.1371/journal.pone.0291925)
Supplement: S1 File — (PDF) [file pone.0291925.s001.pdf]

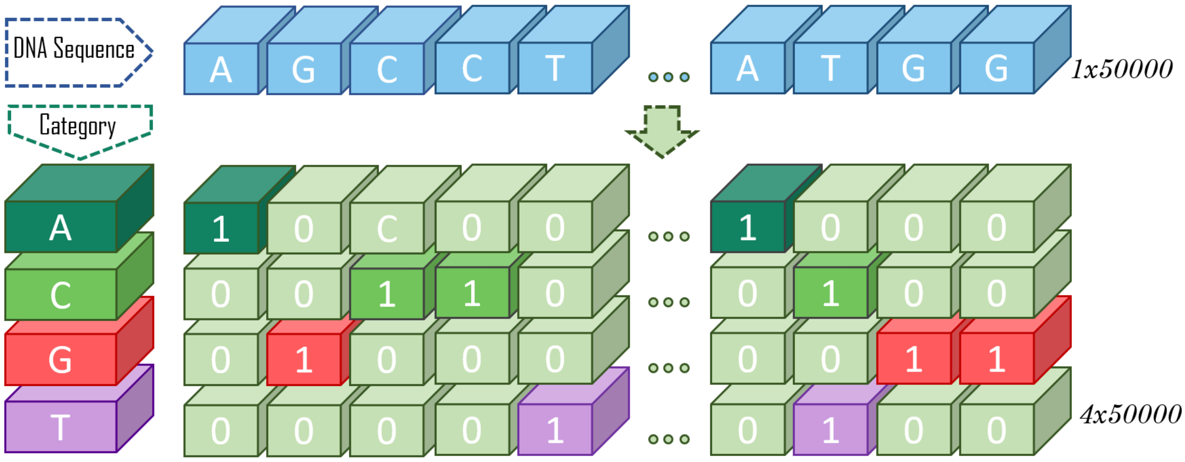

Figure S1. Transformation of DNA sequence information by applying one-hot encoding.

A)

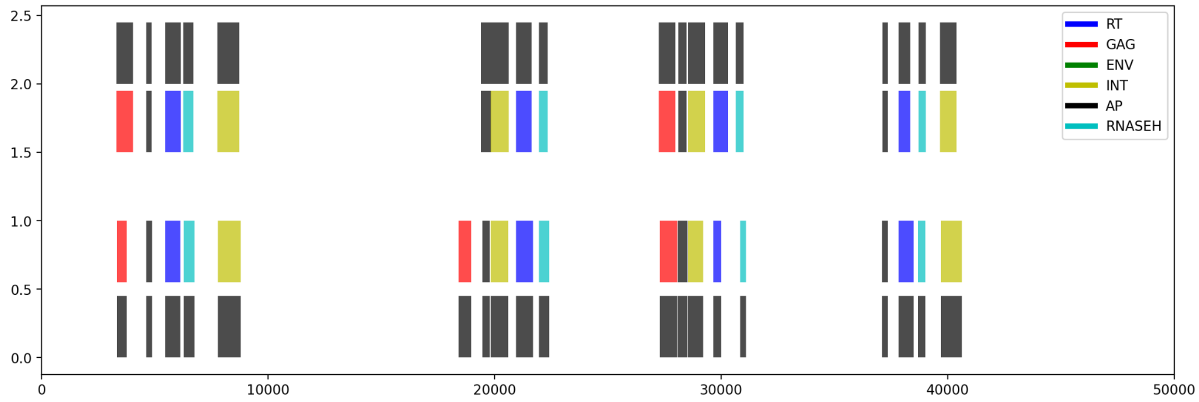

B)

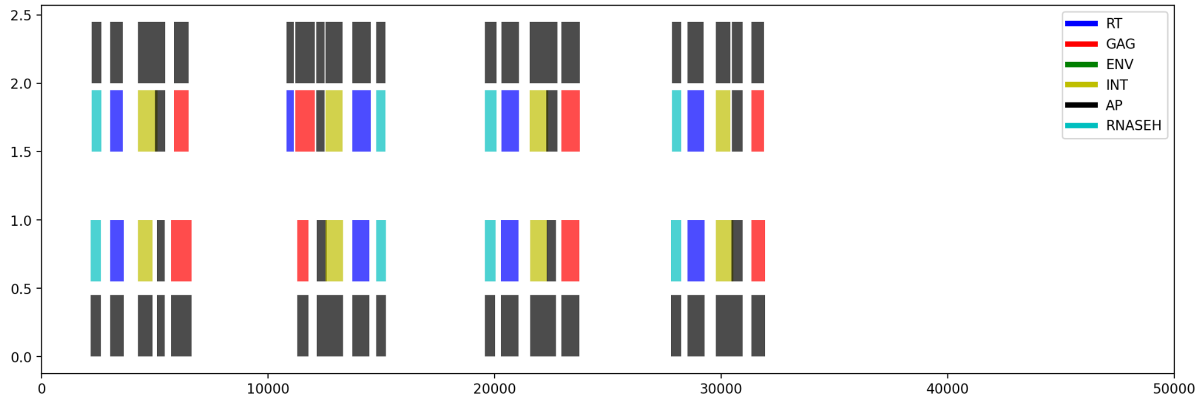

C)

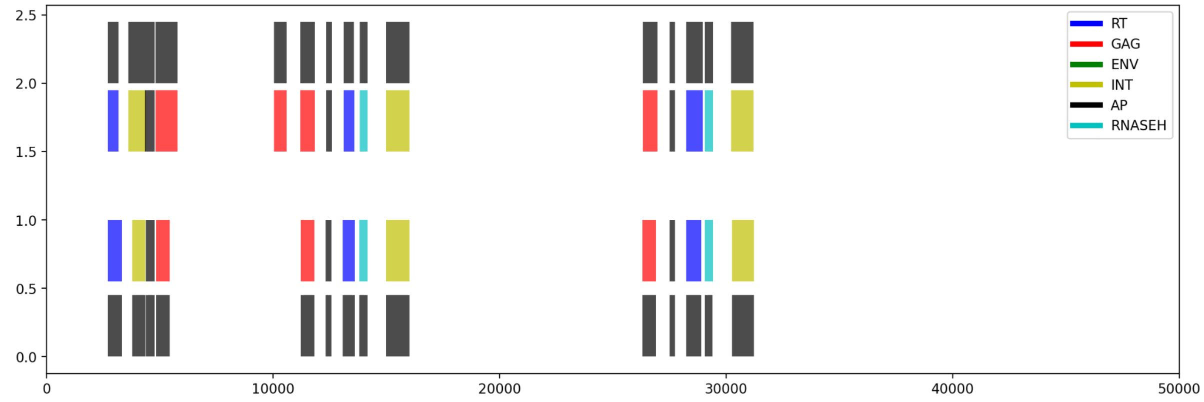
2.5
2.0
1.5
1.0
0.5
0.0
0
10000
20000
30000
40000
50000
RT
GAG
ENV
INT
AP
RNASEH

D)

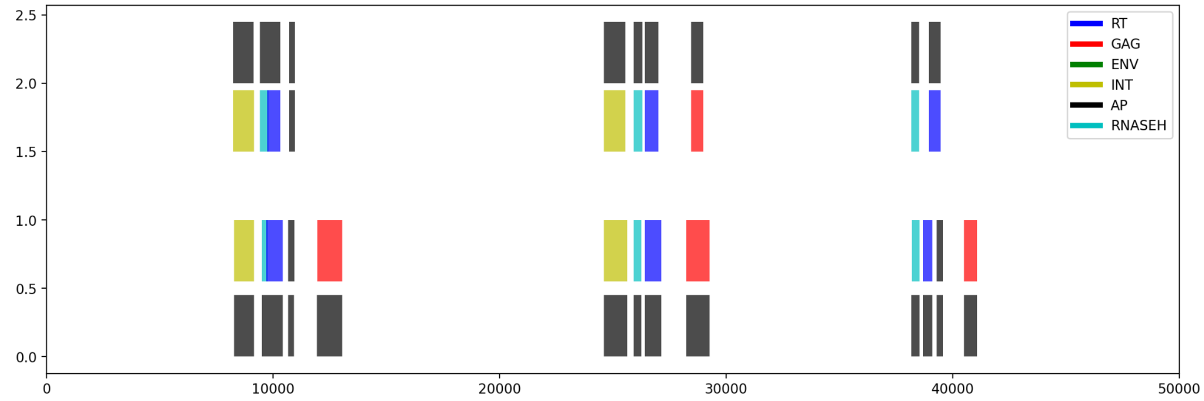
2.5
2.0
1.5
1.0
0.5
0.0
0
10000
20000
30000
40000
50000
RT
GAG
ENV
INT
AP
RNASEH

E)

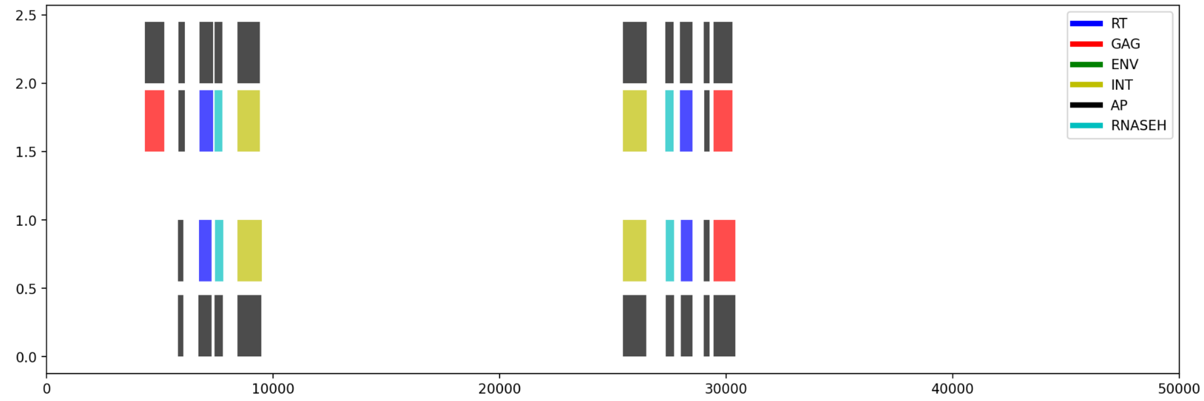
2.5
2.0
1.5
1.0
0.5
0.0
0
10000
20000
30000
40000
50000
RT
GAG
ENV
INT
AP
RNASEH

F)

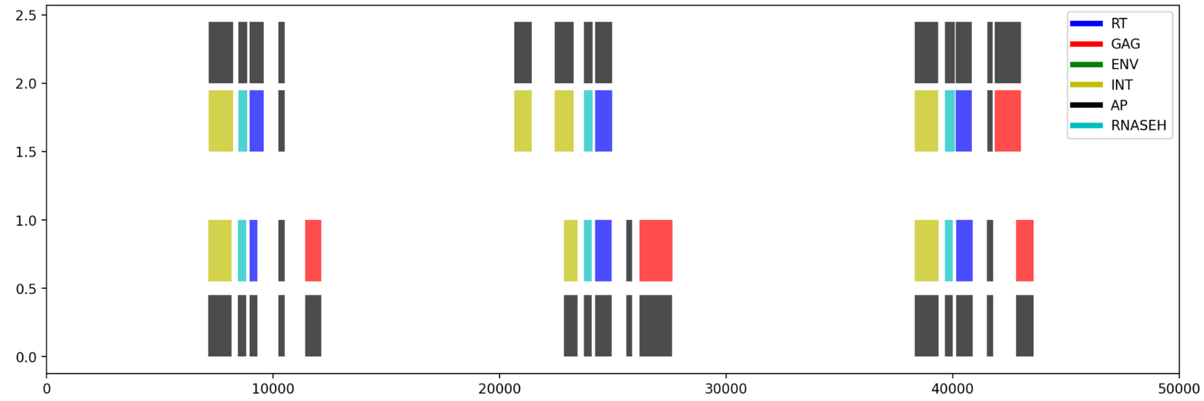
2.5
2.0
1.5
1.0
0.5
0.0
0
10000
20000
30000
40000
50000
RT
GAG
ENV
INT
AP
RNASEH

G)

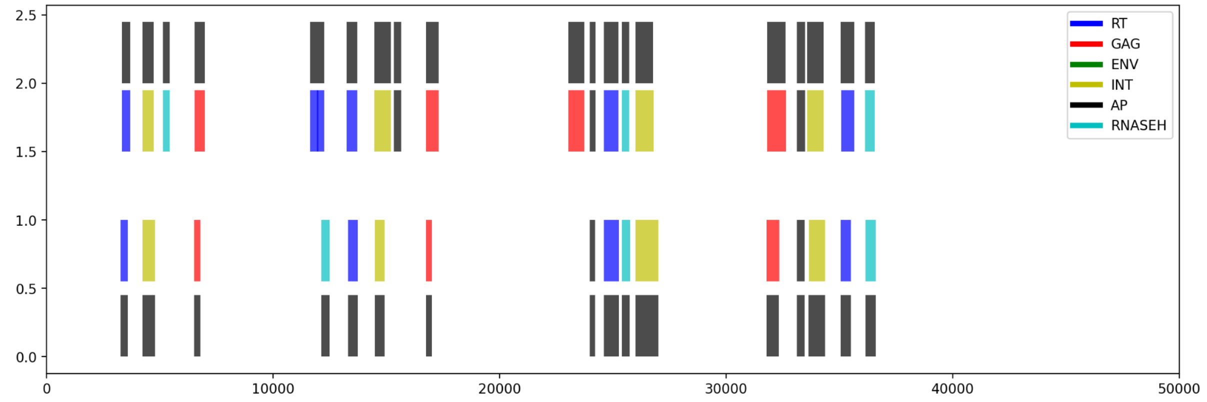
RT
GAG
ENV
INT
AP
RNASEH
2.5
2.0
1.5
1.0
0.5
0.0
0
10000
20000
30000
40000
50000

H)

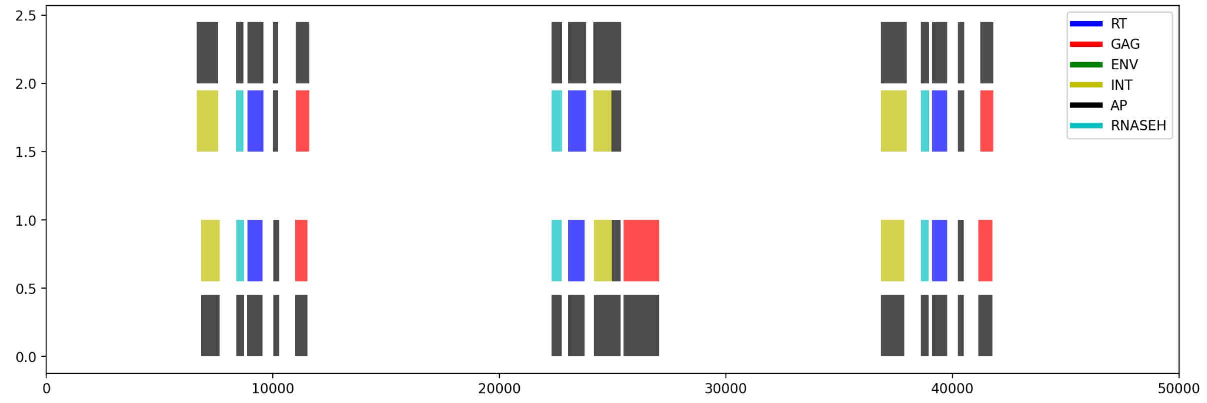
RT
GAG
ENV
INT
AP
RNASEH
2.5
2.0
1.5
1.0
0.5
0.0
0
10000
20000
30000
40000
50000

I)

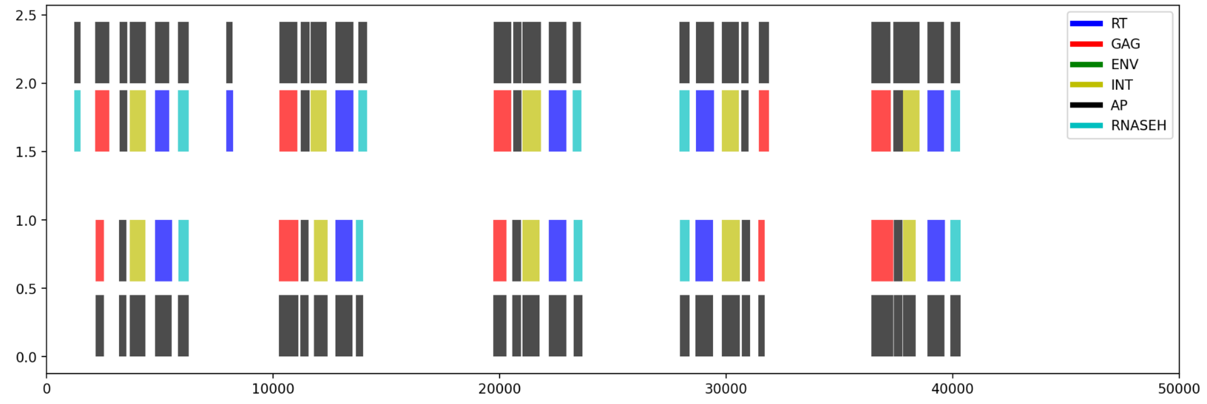
RT
GAG
ENV
INT
AP
RNASEH
2.5
2.0
1.5
1.0
0.5
0.0
0
10000
20000
30000
40000
50000

J)

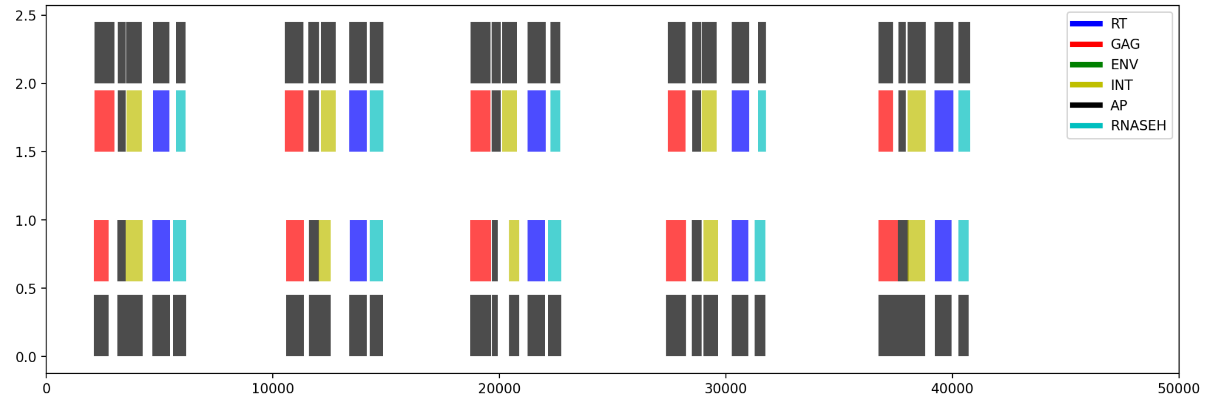
RT
GAG
ENV
INT
AP
RNASEH
2.5
2.0
1.5
1.0
0.5
0.0
0
10000
20000
30000
40000
50000

K)

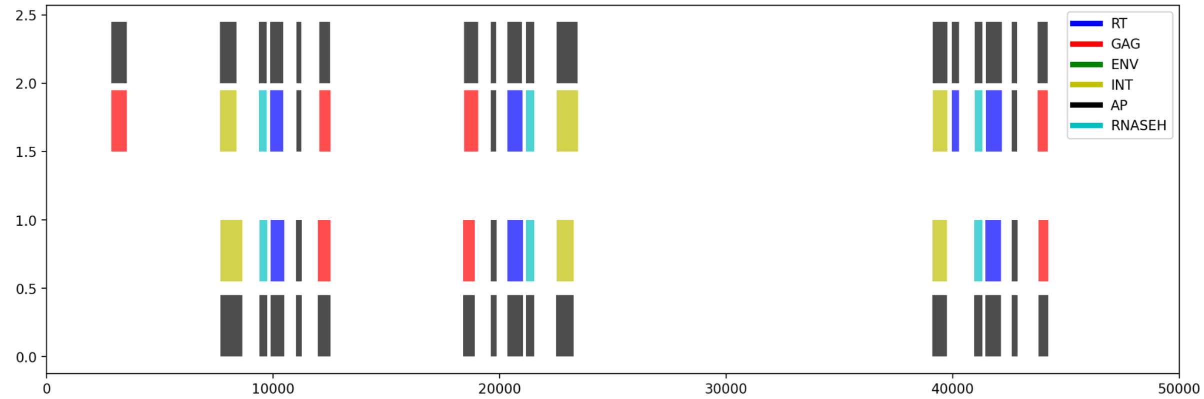
RT
GAG
ENV
INT
AP
RNASEH
2.5
2.0
1.5
1.0
0.5
0.0
0
10000
20000
30000
40000
50000

L)

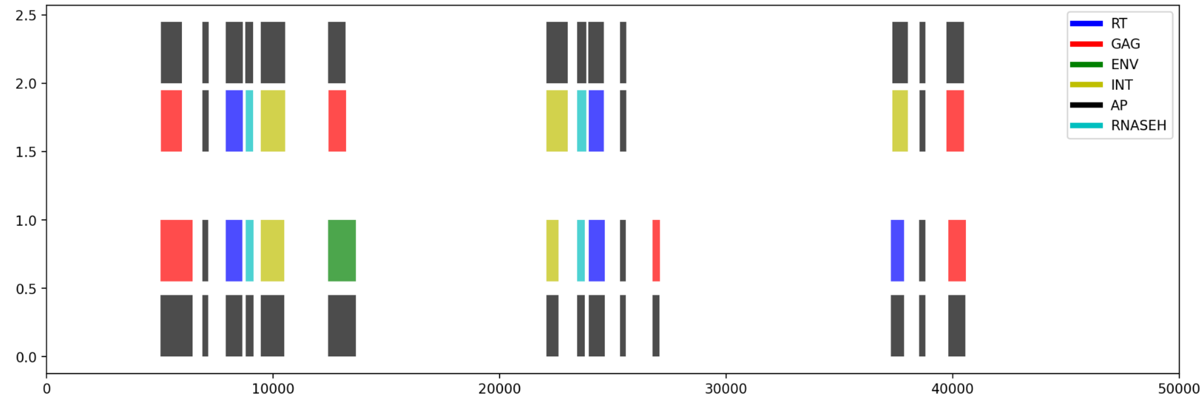
RT
GAG
ENV
INT
AP
RNASEH
2.5
2.0
1.5
1.0
0.5
0.0
0
10000
20000
30000
40000
50000

M)

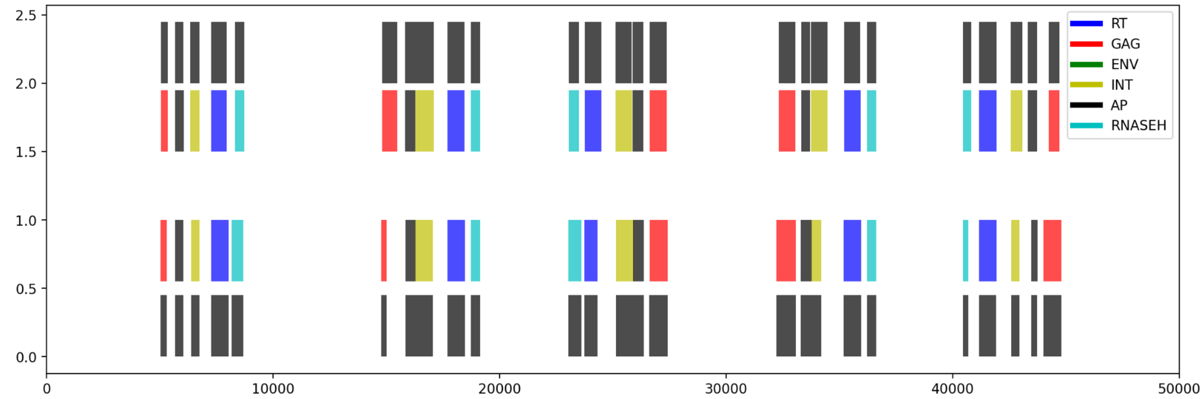
RT
GAG
ENV
INT
AP
RNASEH
2.5
2.0
1.5
1.0
0.5
0.0
0
10000
20000
30000
40000
50000

N)

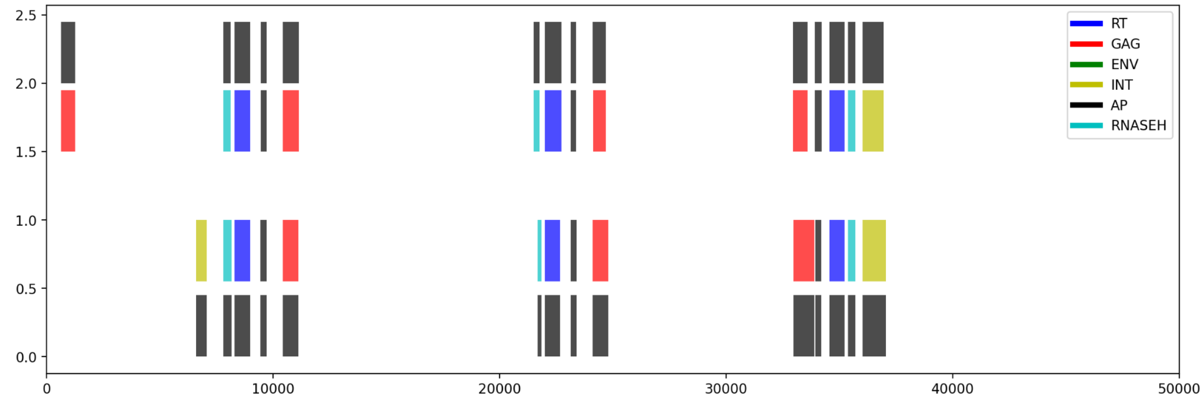
RT
GAG
ENV
INT
AP
RNASEH
2.5
2.0
1.5
1.0
0.5
0.0
0
10000
20000
30000
40000
50000

O)

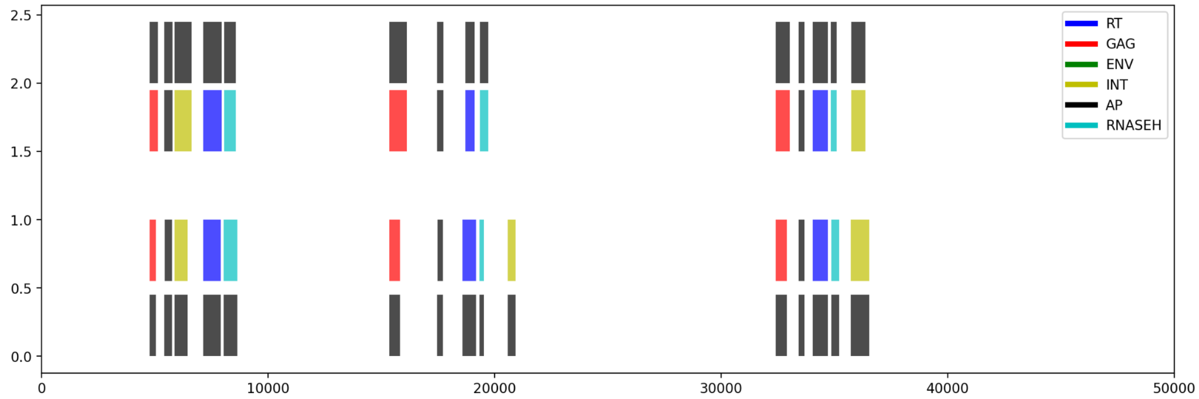

Figure S2. Graphical representation of real positions (lower section) and YORO predictions (upper section) of 15 sequences of 50 kb of length with LTR-retrotransposons inserted, each one separated by each other with negative background. For the negative background we used sequences from DOI 10.5281/zenodo.4543904 that correspond to any plant DNA sequences that are not LTR-RTs such as CDS, RNAs, TEs from other orders, among others. The lineages of the LTR-retrotransposons from left to right are:

A) (1) TAT (2) ORYCO/IVANA (3) TORK/TAR (4) TAT

B) (1) ALE/RETROFIT (2) TORK/TAR (3) ALE/RETROFIT (4) ALE/RETROFIT

C) (1) TORK/TAR (2) TAT (3) TAT

D) (1) ATHILA (2) ATHILA (3) ATHILA

E) (1) TAT (2) TAT

F) (1) ATHILA (2) ATHILA (3) ATHILA

G) (1) BIANCA (2) BIANCA (3) REINA (4) ALE/RETROFIT

H) (1) TAT (2) SIRE (3) TAT

I) (1) ORYCO/IVANA (2) ORYCO/IVANA (3) ORYCO/IVANA (4) ORYCO/IVANA (5) ORYCO/IVANA

J) (1) ALE/RETROFIT (2) ALE/RETROFIT (3) ALE/RETROFIT (4) ALE/RETROFIT (5) TORK/TAR

K) (1) TAT (2) TAT (3) TAT

L) (1) ATHILA (2) ATHILA (3) ATHILA

M) (1) ANGELA (2) ALE/RETROFIT (3) ALE/RETROFIT (4) ALE/RETROFIT (5) ALE/RETROFIT

N) (1) TAT (2) TAT (3) DEL/TEKAY

O) (1) ANGELA (2) TAT (3) DEL/TEKAY

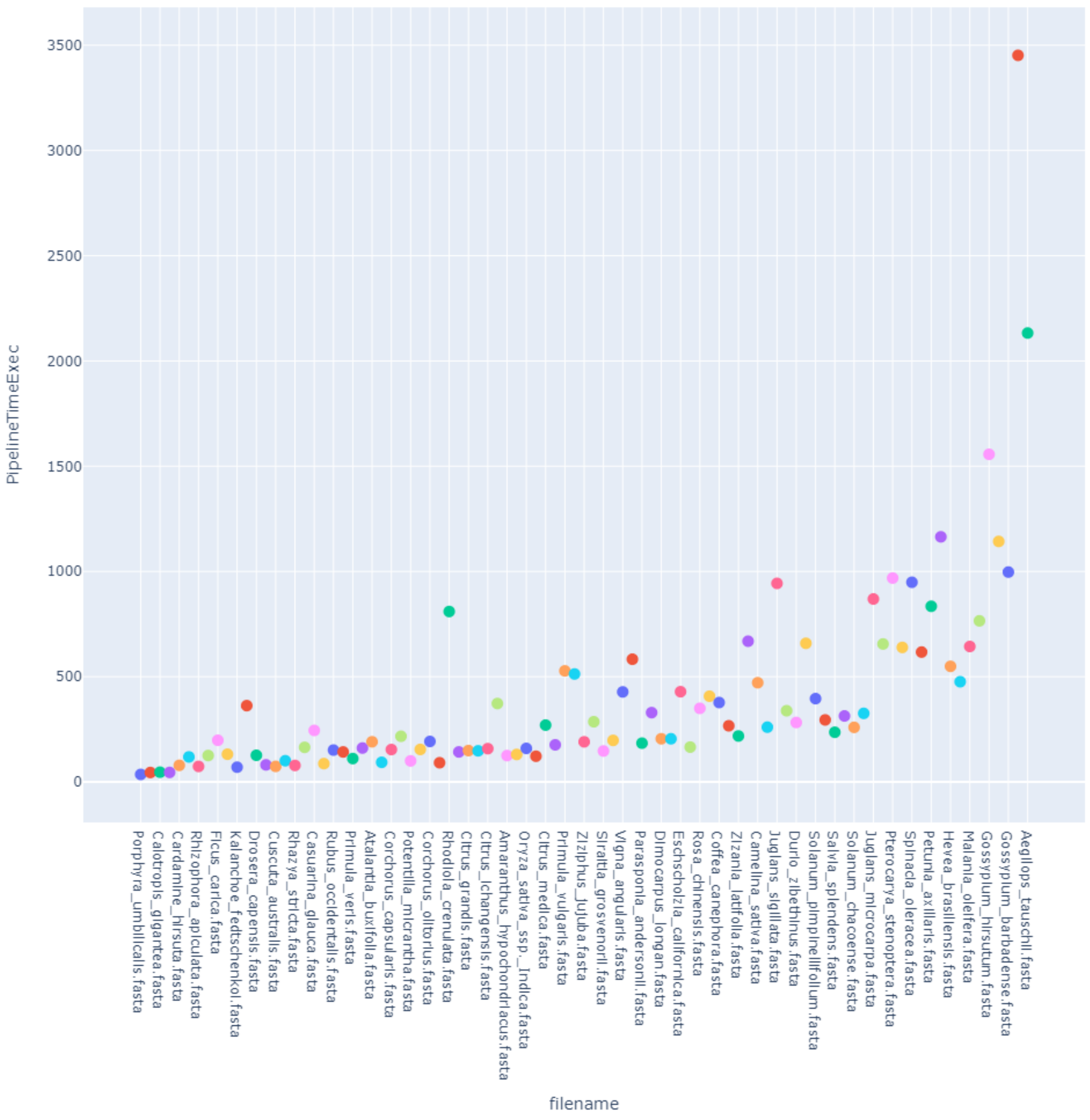

Figure S3. Genomic Object Detection pipeline execution times for 91 plant genomes

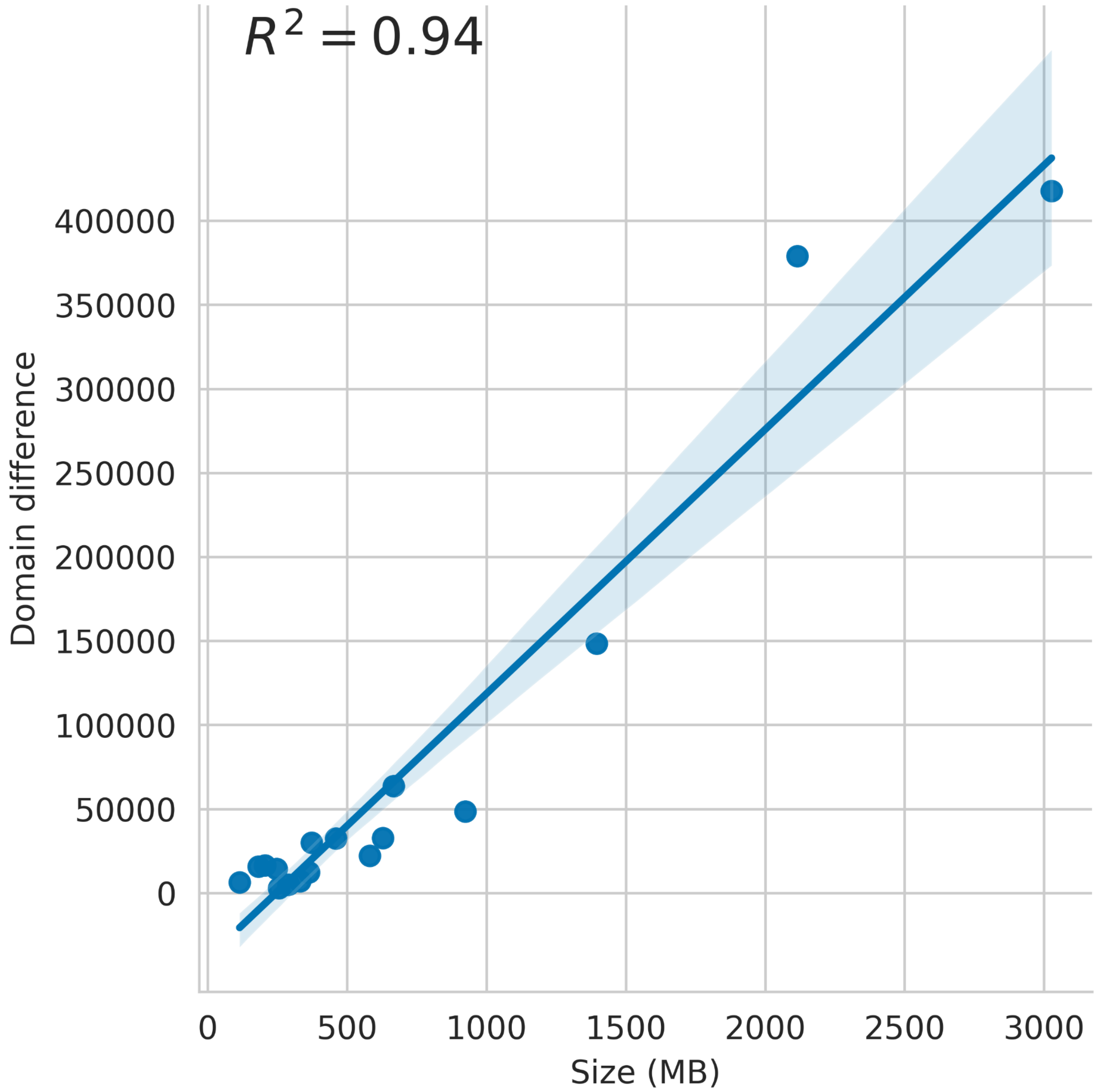

Figure S4. Correlation graph between the difference in the number of domains identified by YORO and BLAST as a function of the size of the genomes analyzed (sample: 17 genomes).

Table S1. Species and sizes used for the execution of BLASTx and YORO.

Plant species	Size (Mbp)	Normalized N50
Arabidopsis thaliana	115.0	0.196
Boechera stricta	182.0	0.141
Selaginella moellendorffii	206.0	0.008
Kalanchoe fedtschenkoi	248.0	0.009
Drosera capensis	256.0	0.000

Selaginella tamariscina	291.0	0.001
Thlaspi arvense	332.0	0.0000
Amaranthus hypochondriacus	364.0	0.000
Oryza sativa ssp. Indica	373.0	0.081
Parasponia andersonii	460.0	0.001
Juglans cathayensis	582.0	0.000
Juglans regia	629.0	0.000
Datisca glomerata	667.0	0.001
Brassica juncea	924.0	0.04
Jaltomata sinuosa	1395.0	0.000
Gossypium hirsutum	2115.0	0.032
Saccharum spontaneum	3027.0	0.029
